# Screening of Cucumber Fusarium Wilt Bio-Inhibitor: High Sporulation *Trichoderma harzianum* Mutant Cultured on Moso Bamboo Medium

**DOI:** 10.3389/fmicb.2021.763006

**Published:** 2021-12-31

**Authors:** Ning Zhang, Hao Xu, Jingcong Xie, Jie-yu Cui, Jing Yang, Jian Zhao, Yajuan Tong, Jianchun Jiang

**Affiliations:** ^1^Institute of Chemical Industry of Forest Products, Chinese Academy of Forestry, National Engineering Laboratory for Biomass Chemical Utilization, Key Laboratory of Chemical Engineering of Forest Products, National Forestry and Grassland Administration, Key Laboratory of Biomass Energy and Material, Nanjing, China; ^2^Co-Innovation Center of Efficient Processing and Utilization of Forest Resources, Nanjing Forestry University, Nanjing, China; ^3^College of Chemical Engineering, Nanjing Forestry University, Nanjing, China

**Keywords:** moso bamboo, *Trichoderma harzianum*, cucumber fusarium wilt, microwave mutagenesis, mutagenesis

## Abstract

Cucumber fusarium wilt is a soil-borne disease which causes serious production decrease in cucumber cultivation world widely. Extensive using of chemical pesticides has caused serious environmental pollution and economic losses, therefore, it is particularly urgent to develop efficient, safe and pollution-free biopesticide. In this study, a mutant strain of *Trichoderma harzianum* cultivated in moso bamboo medium was proved to be an efficient bio-inhibitor of the disease. The mutant strain *T. harzianum* T334, was obtained by three microwave mutagenesis cycles with an irradiation power of 600 W and irradiation time of 40 s. In contrast to the original strain, the inhibition rate on cucumber fusarium wilt of the strain T334 increased from 63 to 78%. In this work, disk milling pretreatment of moso bamboo has shown significant beneficial effects on both biotransformation and sporulation of T334. Its sporulation reached 3.7 × 10^9^ cfu/g in mushroom bags with 90% bamboo stem powder (pretreated by disk milli), 9.5% bamboo leaf powder and 0.5% wheat bran when the ratio of solid to liquid was 4:6, the inoculum amount was 10%, and the culture temperature was 28°C. These results provide an alternative bioinhibitor for the control of cucumber fusarium wilt, and a potential usage of moso bamboo in the production of microbial pesticide.

## HIGHLIGHTS

- Cucumber fusarium wilt inhibition rate of T334 was increased by microwave mutagenesis.
- Sporulation of T334 was increased after microwave mutagenesis.
- Biotransformation rate of moso bamboo was improved after disk milling pretreatment.
- Bamboo stem and leaves are suitable for fermenting production of the mutant T334.

## Introduction

Cucumber fusarium wilt (Jia et al., 2015) is one of the most difficult diseases to control in cucumber cultivation. It is world widely known as “plant cancer,” which is a systemic disease transmitted by soil and invades the root or root neck (Gao et al., 2020). Cucumber fusarium wilt spreads quickly and it only takes 2–3 years from sporadic onset to large-scale onset. The incidence rate can be as high as 70%, and the yield loss is 10–50% or even no harvest in the 3 years of continuous cropping in the same plot. The disease not only harms cucumber, but also harms watermelon, melon, winter melon and so on. The pathogen of Cucumber fusarium wilt is *Fusarium oxysporum. f. sp. cucumebrium*, which overwinters in soil, and diseased plant residues and seeds with mycelium, sclerotium and thick cucumebrium spores become the source of infection at the beginning of the next year and can be transmitted over a long distance with the help of rain and irrigation water (Li et al., 2016). At present, the prevention and control techniques for Cucumber fusarium wilt mainly include selection of disease-resistant varieties, avoidance of continuous cropping, grafting and application of chemical pesticides. Although the extensive use of chemical pesticides has controlled the disease, it has, at the same time, killed the beneficial microorganisms in the environment, seriously damaged the agricultural ecosystem and caused environmental pollution. In addition, the resistance of pathogens increases year by year, which has resulted in a high incidence rate of the disease and difficulty in its prevention (Jia et al., 2015). Therefore, it is particularly urgent to develop high-efficiency, safe and pollution-free green biological pesticides (Cao et al., 2011).

Research on the biological control of Cucumber fusarium wilt has mainly focused on fungi, actinomycetes and bacteria (José et al., 2015). Among them, *T. harzianum* has been attracting the interest of many researchers because it can inhibit plant pathogenic fungi, promote plant growth and induce plant defense through its hyperparasitism and production of antibiotics, including polyketones, amino acids and their derivatives, terpenes, imidazoles, etc. (Khaledi and Taheri, 2016). Although the fermentation technology of *T. harzianum* has been significantly improved, and some products have been put into the market, the backward production technology and the low control effect compared with chemical pesticides limit its wide application. Increment in control effect can be achieved by using microbial genetic breeding techniques including mutation breeding, crossbreeding, metabolic control breeding and genetic engineering breeding etc. By comparison, advanced physical mutagenesis techniques, such as microwave (Sarkar et al., 2015) and low energy ion implantation (Wu et al., 2012; Liu et al., 2015), provide more and more possibilities in parameter selection and control. Combined with sensitive screening factors, they have been well applied in industrial microbial genetics and breeding (Xu et al., 2010; Yan et al., 2014).

On the other hand, the key to the application of *T. harzianum* in agricultural production lies in the number of spores. Fungicides with a high number of spores can germinate and propagate rapidly in the field, form dominant colonies, and then produce metabolites that act on the plant and its growth microenvironment, exerting biological control and promoting plant growth (Yücel et al., 2017). In addition, if the number of spores is high, the shelf life of the fungicide will be longer. At present, wheat bran is the main component in the medium for *T. harzianum* cultivation. The problem with wheat bran is that mycelial growth is too vigorous, and spores are insufficient during the process of cell growth. Therefore, it is key to improve sporulation and develop a culture medium that is suitable for the growth of *T. harzianum*.

Previous studies have found that bamboo powder can be used for the culture of *T. harzianum*, and a selective medium composed of oxygen carrier powder and bamboo powder for *T. harzianum* culture was invented (Qin et al., 2016). However, further analysis of the effect of the pretreatment method on the fibers and lignocellulosic composition after fermentation are still required to determine the bamboo potential for *T. harzianum* production. The analysis results showed that bamboo contains 25–45% cellulose, 20–25% hemicellulose and 20–30% lignin. Compared with straw and wheat straw, bamboo shows little difference in cellulose content but higher lignin content. Compared with hardwood and cork, bamboo has basically the same lignin content but lower cellulose and hemicellulose content (Singh et al., 2016). Therefore, research on biotransformation with bamboo as the substrate has a certain uniqueness. Like other lignocelluloses, the resistance of bamboo fiber to enzymatic hydrolysis is also the bottleneck of bamboo fiber biotransformation (Yang et al., 2011). It is difficult to reduce the crystallinity of cellulose and increase the specific surface area and porosity of substrate as much as possible. Papermaking beating treatment is a process in which fiber is cut off and disported by the mechanical action of beating equipment. Beating treatment can produce a series of effects, such as deformation, wetness swelling, fibrillation and cutting (Salgueiro et al., 2016). As a technology for papermaking beating treatment, the disk mill involves a continuous beating process. This strategy reduces the particle size and increases the specific surface area, thereby improving the biological accessibility of raw materials (Liu et al., 2016).

In this paper we applied the technique of microwave to irradiate *T. harzianum* and selected a mutant with higher inhibition rate on cucumber fusarium wilt. Fatality and mutation rate of different operation parameters of microwave mutagenesis (microwave powers and times) were tested, and then genetic stability of the mutant was investigated. Moso bamboo stem powder, which was pretreated by disk milling, and moso bamboo leaves were used as the medium to ferment *T. harzianum* mutant. The microstructure (IR, XRD, and SEM) of moso bamboo after pretreatment and fermentation were also tested.

## Materials and Methods

### Materials

Moso bamboo powder was supplied by the China National Bamboo Research Center. Moso bamboo leaves were collected from Nanjing Forestry University. *Trichoderma harzianum* (CMCC 3.5488) was purchased from the China General Microbiological Culture Collection Center. The pathogenic strain, *Fusarium oxysporum f. sp. cucumerinum* (CICC 2532) was purchased from the China Center of Industrial Culture Collection.

### Pretreatment Method for Moso Bamboo Materials

The moso bamboo powder was dried to a constant weight and then milled five times by a FSP-300 high-concentration experimental disk mill. The screw rotation speed was 280 rpm, the water supply pressure was 0.5 MPa, the cooling water flow was 2 L/min, and the dilution water flow was 3 L/min (Romuli et al., 2015). The freeness of the obtained material reached 170 ∼ 180 mL. The fresh moso bamboo leaves were dried to constant weight, ground and passed through a 0.178 mm sieve.

### Medium

Solid medium (mass per 1,000 mL of medium): glucose 20 g, KH_2_PO_4_ 3 g, MgSO_4_.7H_2_O 1.5 g, (NH_4_)_2_SO_4_ 0.75 g, wheat bran decoction (Cavalcante et al., 2008) (100 g of bran, 900 g of water, boiling for 20 min, filtered through four layers of gauze) 80 g, agar 15 g. Separate medium formula (mass per 1,000 mL of medium): 1.5 g of sodium deoxycholate was added to the solid medium; liquid medium (mass per 1,000 mL of medium): glucose 20 g, KH_2_PO_4_ 3 g, MgSO_4_.7H_2_O 1.5 g, (NH_4_)_2_SO_4_ 0.75 g, bran water 80 g. Solid fermentation medium: Moso bamboo pretreated with 90 g of disk mill material, 9.5 g of moso bamboo leaf powder, 0.5 g of bran, and 150 mL of liquid medium.

### Fermentation Method

Seed liquid fermentation method (Gu et al., 2008): Appropriate amounts of spores of *T. harzianum* were inserted into liquid medium. The liquid filling quantity was 50 mL in a 250 mL triangular bottle, the rotation speed was 180 r/min, and the culture time was 48 h at 28°C. Solid-state fermentation method: The fermentation medium was placed in a 1,000 mL beaker or mushroom culture bags of 20 cm × 28 cm, sterilized by moist heat, cooled to room temperature, inserted into 5% *T. harzianum* seed liquid, and placed in a 28°C incubator for 7–10 days. After 48 h of inoculation, the culture medium and mycelium were mixed evenly every 48 h, and an appropriate amount of sterile water was added until the end of the fermentation.

### Breeding of *T. harzianum* by Microwave Mutation

#### Microwave Mutagenesis Method

Microwave mutagenesis was carried out with MWD-520 microwave digestion system which was produced by Shanghai Metash Instruments Co., Ltd. Physiological saline was added to the separation medium of *T. harzianum*, and the spores were washed and poured into a sterilized triangular flask equipped with a magnetic stirrer to disperse the spores for use (Osula et al., 2015). The concentration of spore suspension was controlled at 10^7^∼10^8^ cfu/mL. The spore suspension was transferred into a 1.5 mL centrifuge tube, and the centrifuge tube was placed into a microwave digestion tube containing ice cubes (Jia et al., 2003). The spore suspension was irradiated with power levels of 200, 400, 600, 800, and 1,000 W, and the treatment time was 0 ∼ 180 s (Li, 2001). Then, the spore suspension was coated on the separation medium plate, and the number of colonies was counted after incubation at 28°C for 2 days to calculate the lethal rate of microwaves under different irradiation powers and times, according to the equation (1).


(1) $$\mathrm{Lethal}\mathrm{rate}\left(\%\right)=\frac{\left(N_{0}-N_{c}\right)}{N_{0}}\times 100$$

*N*_0_ — Colony number of coated plate without microwave irradiation;

*N*_*c*_ — Colony number of the coated plate after microwave irradiation for different times.

#### Screening Method of the *T. harzianum* Mutant

Primary screening of mutant strains (point confrontation experiment): *Fusarium oxysporum. f. sp. cucumebrium* and irradiated *T. harzianum* and were inoculated symmetrically on solid medium at the same time (Karkachi et al., 2012). The distance between them was approximately 3 cm. The plates that were only inoculated with *Fusarium oxysporum. f. sp. cucumebrium* were used as a control. Then, the diameters of *Fusarium oxysporum. f. sp. cucumebrium* on the experimental and the control plates were measured regularly. The inhibition rate of *T. harzianum* on *Fusarium oxysporum. f. sp. cucumebrium* was calculated according to the equation (2).


(2) $$Inhibitionrate\left(\%\right)=\frac{\left(D_{0}-D_{c}\right)}{D_{0}}\times 100$$

*D*_0_ — Diameter of the colony without microwave irradiation;

*D*_*c*_ — Diameter of the colony after microwave irradiation.

Compared with the original strain, the mutant was defined as positive if the inhibition rate was confirmed to be a 5% increase after incubation; inversely, the mutant was defined as negative if a 5% decrease was confirmed.

Secondary screening of mutant strains: the mutant strain with a significantly increased inhibition rate was selected, inserted into the solid fermentation medium at a 5% inoculation volume, and placed in a 28°C incubator for 7 days. In this way, mutant strains with high antibacterial efficiency and high spore yield were selected.

#### Genetic Stability

To ensure the genetic stability of the mutant strains, the genetic stability was tested. The inhibition rate and sporulation after solid fermentation were detected. *T. harzianum* was mutagenized three times and passaged five times, and three parallel samples were made in each generation.

### Analytical Methods

#### Determination of the Number of Spores

Solid culture (0.5 g) was diluted 100 times with sterile water containing 1% Tween, stirred on a magnetic stirrer for 20 min, and the number of spores was counted under a microscope with a hemocytometer.

#### Infrared Spectrum Analysis

**A** Nicolet iS10 Fourier infrared spectrometer produced by Thermo Nicolet Corporation was used to detect the structural changes of the samples. The scanning wavenumber range was 4,000–500 cm^–1^. The calculation method of the relative intensity of the absorption peak was the ratio of the absorbance of the corresponding characteristic wavenumber to the absorbance of 1,372 cm^–1^ (Chandel et al., 2014). The experimental results were the average of three experimental repeats.

#### X-Ray Diffraction Analysis

**A** D8 Foucus X-ray diffractometer (XRD) produced by Bruker Corporation was used to determine the relative crystallinity of the samples. The detection wavelength was 0.15406 nm, and the sampling interval was 0.02°. Then, the crystallinity was calculated according to the equation (Segal et al., 1959). The experimental results were the average of three experimental repeats.


(3) $$Crl\left(\%\right)=\frac{\left(I_{002}-I_{am}\right)}{I_{002}}\times 100$$

*Crl*(%) — The percentage of relative crystallinity;

*I*_002_ — The maximum intensity (arbitrary unit) of the lattice diffraction angle;

*I*_*am*_ — The scattering intensity of the non-crystalline background diffraction when the 2θ angle is close to 18°.

#### Scanning Electron Microscope Analysis

Observation was carried out with a 3400-I scanning electron microscope (SEM) produced by Hitachi Limited. The Moso bamboo raw material, the raw material after pretreatment with a disk mill, and the culture medium after fermentation were observed at 1,000 times and 2,000 times magnification, respectively.

## Results and Discussion

### Breeding of *T. harzianum* Mutants by Microwave Irradiation

#### Mutagenic Effects of *T. harzianum* by Microwave Irradiation

**FIGURE 1:**
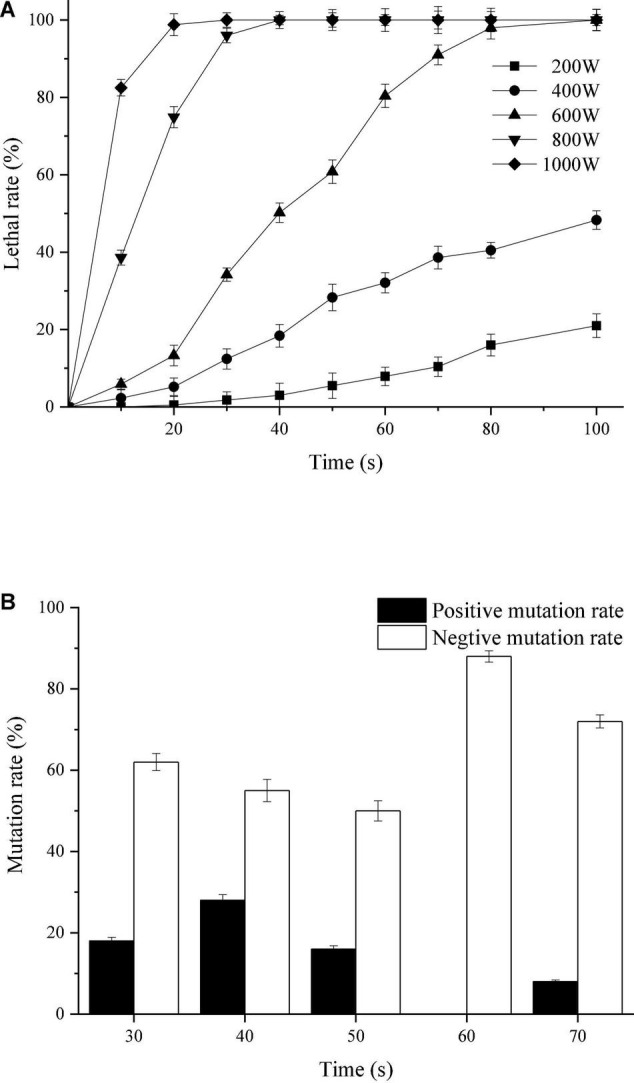
Mutagenic effects of *T. harzianum* by microwave irradiation. **(A)** Lethal rate of *T. harzianum* irradiated by microwaves of different powers (200, 400, 600, 800, and 1,000 W). **(B)** Mutation rate of *T. harzianum* irradiated by microwaves of different times (30, 40, 50, 60, and 70 s) under the irradiated power of 600 W. The experiments were repeated three times.

Existing studies have shown that microwave irradiation can cause damage or weakening of hydrogen bonding and base stacking chemical forces of microbial DNA molecules and change the three-dimensional structure of DNA, which leads to the occurrence of mutations (Mazinani et al., 2017). It can be seen from Figure 1A that the lethal rate of *T. harzianum* increased with the increase of irradiation power and the length of irradiation time; when the irradiation power was 200 W, the lethal rate was less than 20% within 100 s; when the irradiation was 400 W, the lethal rate within 100 s was less than 50%. When the irradiation power was 600 W and the irradiation time was 20 ∼ 60 s, the lethal rate was concentrated at 40 ∼ 80%, while continuing to increase the irradiation power, the lethal rate increased to 100%. According to the repair theory (Stuckey and Storici, 2013), the repair capacity of the cell depends on the irradiation intensity or tends to be saturated with increasing irradiation intensity. The irradiation intensity continued to increase, and the lethal rate was no longer significant, indicating that the repair system of the cell was activated. Of course, if the irradiation intensity exceeded the repair ability of the cell, the strains would die. Therefore, the optimal irradiation time can be determined at the turning point of the lethal rate curve from a sharp increase to a stable level. Based on the lethal situation of *T. harzianum* (Figure 1A), the optimal irradiation power was 600 W, and the irradiation time was 30 ∼ 70 s. At optimal irradiation condition, different mutation rate of *T. harzianum* was exhibited under different irradiation times (Figure 1B). When the irradiation time was 40 s, the positive mutation rate of the strain reached 28%, and the negative mutation was the lowest at 50 s. When the irradiation time was 60 s, no positive mutation was obtained. To select the colony most likely to have a higher sporulation, the subsequent mutagenesis operations were performed with an irradiation time of 40 s.

#### Screening of the *T. harzianum* Mutant and Genetic Stability

**FIGURE 2:**
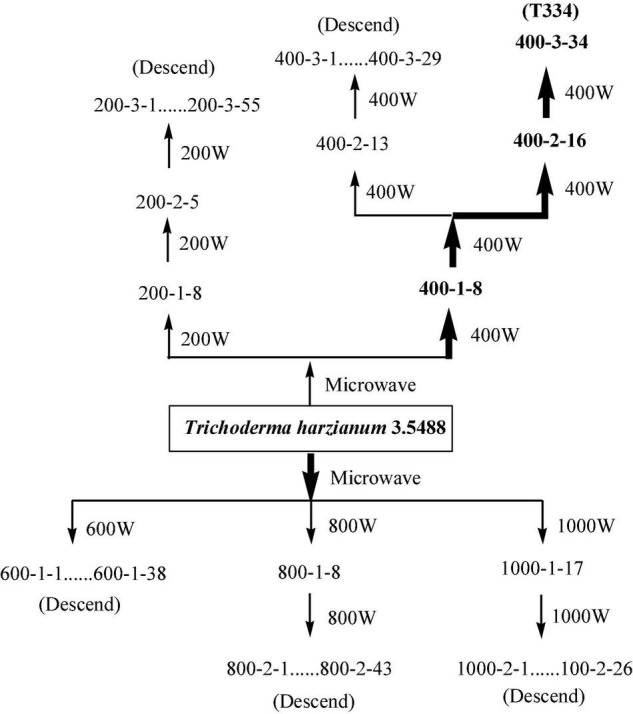
Mutation spectrum of the *T. harzianum* mutant. The first group of numbers in the spectrogram indicates the microwave power, the subsequent numbers indicate the times of microwave irradiation, and then the number of the strain. For example, 200-2-5 represents the 5th mutant strain selected in the second round of mutagenesis under the condition of 200 W.

**FIGURE 3:**
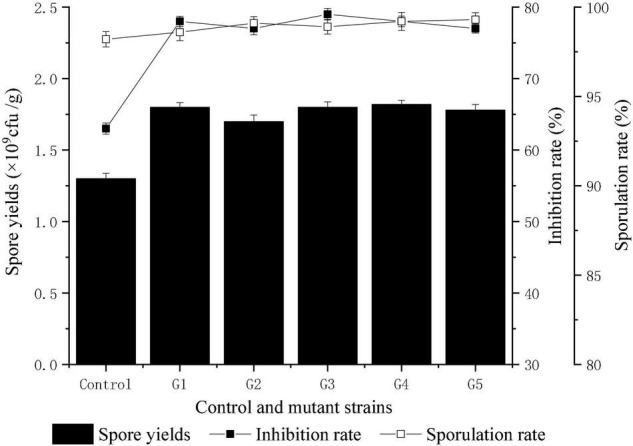
The genetic stability of mutant T334. The comparative experiments were carried out for five consecutive generations with three samples. The results were the average of the three samples. All experiments were carried out as biological duplicates.

Compared with *Fusarium oxysporum f. sp. cucumerinum, T. harzianum* grew rapidly and had competitive advantages in nutrients and space. In this study, the rapid growth of *T. harzianum* was used for mutation screening. Twenty-four hours after inoculation, the colonies of *T. harzianum* were much larger than those of *Fusarium oxysporum f. sp. cucumerinum*, and at 48 h, parts of both colonies began to contact each other gradually After 72 h, an obvious antimicrobial line was formed between the two colonies where they were in contact, and *T. harzianum* began to surround *Fusarium oxysporum f. sp. cucumerinum*. Ninety-six hours later, *T. harzianum* completely surrounded *Fusarium oxysporum f. sp. cucumerinum*. With the increase in the number of spores on both sides, the colonies of *T. harzianum* turned green. These clustered conidia of *T. harzianum* began to be produced on the white colony of *Fusarium oxysporum f. sp. cucumerinum* and increased. Finally, the colony of *Fusarium oxysporum f. sp. cucumerinum* was disintegrated. By measuring the colony diameter of *Fusarium oxysporum f. sp. cucumerinum* after incubation of 96 h, the inhibitory effect of *T. harzianum* on *Fusarium oxysporum f. sp. cucumerinum* was investigated. The mutation screening process is shown in Figure 2. After three microwave mutations, a mutant 400-3-34 (abbreviated as T334) was obtained, and its inhibition rate of *Fusarium oxysporum f. sp. cucumerinum* reached 78% and increased 23.81% compared with the original strain. It can be seen from the growth trend that the mutant T334 grew faster, and spores grew vigorously. Its sporulation increased from 1.3 × 10^9^ cfu/g to 1.8 × 10^9^ cfu/g, and the sporulation and inhibition rate of T334 remained stable after five generations of flask culture. The experimental results are shown in Figure 3. The mutagenesis effect of microwave irradiation on *T. harzianum was* preliminarily studied, the results of existing studies showed that microwave irradiation could cause changes in the activity of antioxidant enzymes in *T. harzianum*, including superoxide dismutase (SOD), peroxidase (POD), catalase (CAT), polyphenol oxidase (PPO) and so on (Kushwah et al., 2013). We speculated that this not only affected the survival rate of *T. harzianum* after mutagenesis, but also may be related to its antimicrobial activity and sporulation (Li et al., 2011).

### Effect of Fermentation Conditions on Spore Production

**FIGURE 4:**
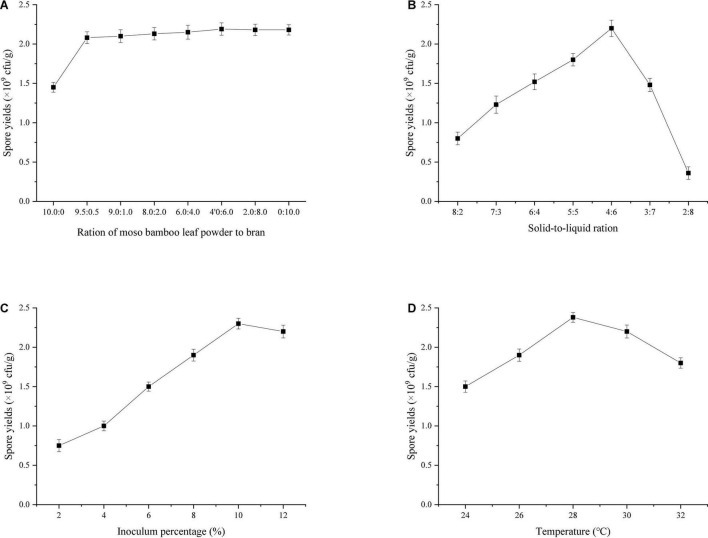
Effects of fermentation conditions on spore production. After each factor was optimized, the optimal condition was substituted into the optimization experiment of the next factor. The experiments were repeated three times. **(A)** Effect of the ratio of moso bamboo leaf to bran on sporulation (10:0, 9.5:0.5, 9.0:1.0, 8.0:2.0, 6.0:4.0, 20:8.0, and 0:10.0). Solid-to-liquid ratio (5:5), inoculum percentage (8%), fermentation temperature (28°C). **(B)** Effect of solid-to-liquid ratio on sporulation (8:2, 7:3, 6:4, 5:5, 4:6, 3:7, 2:8). The ratio of moso bamboo leaf to bran (9.5:0.5), inoculum percentage (8%), fermentation temperature (28°C). **(C)** Effect of inoculum percentage on sporulation (2, 4, 6, 8, 10, and 12%). The ratio of moso bamboo leaf to bran (9.5:0.5), solid-to-liquid ratio (4:6), fermentation temperature (28°C). **(D)** Effect on fermentation temperature on spore production (24, 26, 28, 30, and 32°C). The ratio of moso bamboo leaf to bran (9.5:0.5), solid-to-liquid ratio (4:6), inoculum percentage (10%).

Previous experiments found that 90% bamboo powder was the most suitable medium for the growth of *T. harzianum*. In this study, the ratio of bamboo leaf powder to wheat bran in the remaining 10% was optimized. The results (Figure 4A) showed that the sporulation of T334 increased rapidly when the wheat bran content in bamboo leaf powder increased to 0.5%, but the increase was not significant (the increase was less than 5%) when its content continued to increase, which indicated that 9.5% wheat bran in the medium could be replaced by bamboo leaf powder. Under this condition, the sporulation of T334 was 2.08 × 10^9^cfu/g; the solid-liquid ratio of the medium directly affected the sporulation of T334. The water storage capacity of bamboo powder was weak, and the excess liquid medium accumulated at the bottom of the beaker could not be used. However, if the liquid medium was insufficient, it could not provide enough soluble nutrition for the growth of T334. Repeated observations showed that the sporulation of T334 was highest when the ratio of solid to liquid was 4:6 (see Figure 4B). During the experiment, it was found that there was a strong relationship between the growth of T334 and the inoculation amount. Low inoculation amounts slowed the growth of T334 and prolonged the fermentation cycle. The results showed that sporulation was better when the inoculation amount was 10% (Figure 4C), and sporulation did not increase with increasing inoculation amount. The fermentation temperature experimental results (Figure 4D) showed that the optimal spore producing temperature of T334 was 28°C, and the mycelia could grow well within the experimental temperature range. According to the above fermentation conditions, fermentation experiments were carried out in mushroom culture bags of 20 cm × 28 cm, and the sporulation of T334 could reach 3.72 × 10^9^ cfu/g.

### Effect of Pretreatment and T334 Fermentation on the Chemical Composition and Microstructure of Moso Bamboo

#### Chemical Composition Analysis

After pretreatment by disk milling, the material was dried together with the solution for use, so the total mass loss and the contents of cellulose and lignin did not change much. After the spores were separated after fermentation, the fermentation medium was washed with water to remove the bamboo leaf residues, and the composition changes of the remaining bamboo powder were determined. The results (Table 1) showed that the content of hemicellulose in bamboo powder decreased by 67.59%, and the content of cellulose decreased by approximately 13.77%. Therefore, bamboo stem powder helped to increase the amount of air in the fermentation medium for its loos texture, and provided sufficient carbon source and nutrients for the growth of *T. harzianum*.

**TABLE 1:** Chemical composition change before and after fermentation.

Samples	Solid remain	Cellulose (%)		Hemicellulose (%)		Lignin (%)	
	(%)	Content	removal	Content	removal	Content	removal
Moso bamboo material Before fermentation After fermentation	100.00 96.26 78.51	38.96 38.67 42.79	— 4.46 13.77	27.52 26.04/ 11.36/	— 8.92 67.59	22.24 22.50/ 26.37/	— 2.61 6.91

#### FIR Analysis

The moso bamboo raw material, the pretreated material and the after-fermentation material were scanned by infrared spectroscopy, and the relative absorption intensity in the infrared spectrum is shown in Table 1. After pretreatment by disk milling, the absorption peak intensity of the moso bamboo material at 3,400, 2,900 and 1,432 cm^–1^ decreased (Table 2), which indicated that the intramolecular hydrogen bond, intermolecular hydrogen bond and methylene bond were broken. The absorption peak intensity at 1,163 and 898 cm^–1^ increased (Table 2), which showed that the disk milling treatment was helpful for the depolymerization and separation of cellulose, hemicellulose and lignin but did not destroy the β-glycoside bond and C-O-C bond between glucose and cellulose, which was beneficial to the growth and utilization of microorganisms. After fermentation, the absorption peak intensities at 3,400, 1,432, 1,372, and 898 cm^–1^ were all reduced (Table 2), indicating that the fermentation of T334 caused the hydrogen bond and methylene bond in the molecular structure of moso bamboo to break. The β-glucoside bond of the glucose molecule inside the cellulose molecule and the C-O-C bond of the glucose molecule were destroyed (Liu et al., 2012). The reduction in the absorption strength of the above structure also showed that the growth of T334 consumed a lot of carbon sources, while the absorption peak intensity at 2,900 cm^–1^ increased (Table 2), which indicated that more intermolecular hydrogen bonds were formed in culture during the growth and reproduction of T334, and the formation of intermolecular hydrogen bonds allowed the solute to be better surrounded by water molecules and dispersed into water, which helped the growth and reproduction of T334 (Wang et al., 2020). In addition, T334 could secret cellulase, which in turn contributed to the degradation of culture medium.

**TABLE 2:** Structure attribution and relative intensity change of infrared absorption peak (Kim and Lee, 2005).

Wave length σ/cm^–1^	Response peak	Moso bamboo	After pretreatment	After fermentation
3,400	Intramolecular –OH	2.020	1.999	2.162
2,900	Intermolecular –OH	1.614	1.346	1.509
1,432	Cellulose –CH_2_	1.2625	0.810	0.850
1,372	Cellulose and hemicellulose –CH	1.000	1.000	1.000
1,163	Cellulose and hemicellulose C–O–C	1.701	2.027	1.648
898	β-Glucoside bond	0.687	0.834	0.635

#### X-Ray Diffraction Analysis of Medium

**FIGURE 5:**
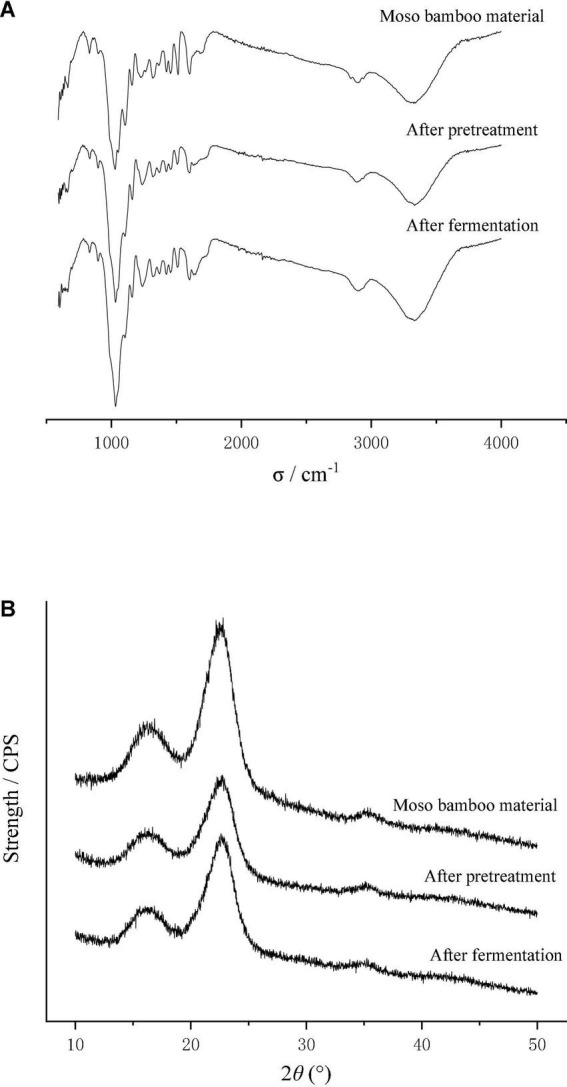
Effect of pretreatment and T334 fermentation on the microstructure of moso bamboo. **(A)** Infrared spectrum of moso bamboo pretreated by disk mill and after fermentation. Samples of the moso bamboo raw material, after pretreatment and after fermentation were scanned with a Nicolet iS10 Fourier Infrared Spectrometer, and the wavelength was 500–4,000 cm^–1^. Each sample was scanned three times, and the result was the average of three times. **(B)** X-ray diffraction pattern of moso bamboo pretreated by disk mill and after fermentation. Samples of the moso bamboo raw material, after pretreatment and after fermentation were scanned with a D8 Foucus X-ray Diffraction instrument. The detection wavelength was 0.15406 nm, and the sampling interval was 0.02°. Each sample was scanned three times, and the result was the average of three times.

Figure 5 shows the X-ray diffraction curves before and after the fermentation of bamboo culture medium, and the changes of crystallinity was calculated according to equation (3). The results showed that the crystallinity of moso bamboo fiber decreased by 30% after disk milling, which indicates that the crystallization area of moso bamboo fiber was destroyed, resulting from the destruction of the intramolecular hydrogen bonds, intermolecular hydrogen bonds and methylene bonds in the bamboo fibers. After fermentation, the crystallinity reached 0.459 and increased 36.0% compared to the material that was pretreated by the disk mill. The reason may be that the growth of T334 mainly resulted in a disordered structure on the surface of the moso bamboo fiber, resulting in a decrease in the amorphous structure and an increase in the crystalline structure (Ju et al., 2015). In the same way, the increase in crystallization of the fermentation substrate also led to the slowing down of the fermentation process to the end. The results of chemical component analysis showed that cellulose and lignin were the main residues in the fermentation substrate after fermentation, and the premise of continuous fermentation was the continuous depolymerization of cellulose and the removal of lignin.

#### Scanning Electron Microscopy of Culture Medium

**FIGURE 6:**
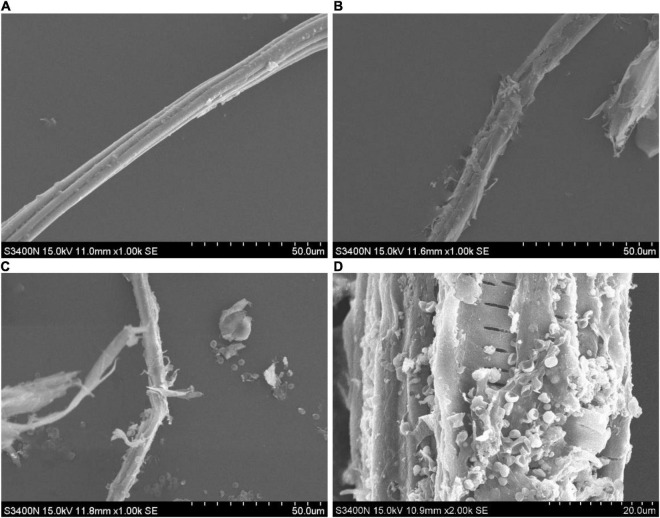
SEM image of moso bamboo culture medium. Samples of the moso bamboo raw material, after pretreatment and after fermentation were scanned using a 3400-I scanning electron microscope. **(A)** Moso bamboo material. **(B)** Material pretreated by disk milling. **(C)** Moso bamboo culture medium after fermentation at 1,000⋄ magnification. **(D)** Moso bamboo culture medium after fermentation at 2,000⋄ magnification.

It can be seen from the scanning electron microscopy that the surface of the moso bamboo raw material was smooth, and the fibers were arranged in order, as shown in Figure 6A. After pretreatment by disk milling, the moso bamboo fibers were obviously broomed and divided into filaments, and such structural changes were undoubtedly conducive to the growth and reproduction of the mycelium, as shown in Figure 6B. After fermentation (Figure 6C), the mycelium of T334 and the bamboo fiber were intertwined, and many spores could be seen inside and outside the sporangium. From 2,000 ⋄magnification (Figure 6D), we can see the orderly arrangement of fiber structure inside. On the other hand, the growth of T334 also contributed to further degradation of the bamboo fiber. The degradation process resulted in degradation of the surface amorphous area, which was more easily degraded first, and then the degradation of the crystallized area with high crystallinity.

## Conclusion

An improved biocontrol agent *T. harzianum* T334 was obtained by microwave mutagenesis. Compared with the original strain, the inhibition rate of the mutated strain against cucumber fusarium wilt increased by 23.81% from 63 to 78%. Moso bamboo was investigated as a new material for fermenting production of the microbial pesticide T334, and whole bamboo utilization was realized with 90% moso bamboo stem powder (pretreated by disk mill), 9.5% moso bamboo leaf powder and 0.5% wheat bran. Under optimal fermentation conditions (solid to liquid 4:6, inoculum 10%, and fermentation at 28°C), the sporulation reached 3.72 × 10^9^ cfu/g when cultured in mushroom bags. After fermentation, the content of hemicellulose in bamboo powder decreased by 67.59%, and the content of cellulose decreased by 13.77%. The crystallinity of bamboo medium increased from 0.337 to 0.459, which indicated that the growth and reproduction of T334 consumed nutrients in the medium of moso bamboo and also changed its microstructure.

## Data Availability Statement

The original contributions presented in the study are included in the article/Supplementary Material, further inquiries can be directed to the corresponding author/s.

## Author Contributions

JJ and JX conceived and designed the experiments. NZ, HX, JX, and J-YC performed the experiments. NZ, JY, and JZ analyzed the data. HX, JY, YT, and JZ contributed to the reagents, materials, and analysis tools. NZ wrote the manuscript. JJ, NZ, and JX revised and approved the final version of the manuscript. All authors contributed to the article and approved the submitted version.

## Conflict of Interest

The authors declare that the research was conducted in the absence of any commercial or financial relationships that could be construed as a potential conflict of interest. The reviewers ZL and XZ declared a shared affiliation with the author, J-YC, to the handling editor at the time of review.

## Publisher’s Note

All claims expressed in this article are solely those of the authors and do not necessarily represent those of their affiliated organizations, or those of the publisher, the editors and the reviewers. Any product that may be evaluated in this article, or claim that may be made by its manufacturer, is not guaranteed or endorsed by the publisher.

## References

[B1] Cao Y. Zhang Z. Ling N. Yuan Y. Zheng X. Shen B. (2011). Bacillus subtilis SQR 9 can control fusarium wilt in cucumber by colonizing plant roots. *Biol. Fertil. Soils.* 47 495–506. 10.1007/s00374-011-0556-2

[B2] Cavalcante R. S. Lima H. Pinto G. A. Gava C. A. Rodrigues S. (2008). Effect of moisture on *Trichoderma* conidia production on crn and wheat bran by solid State fermentation. *Food Bioproc. Tech.* 1 100–104. 10.1007/s11947-007-0034-x

[B3] Chandel A. K. Antunes F. A. Anjos V. Bell M. J. Rodrigues L. N. Polikarpov I. (2014). Multi-scale structural and chemical analysis of sugarcane bagasse in the process of sequential acid–base pretreatment and ethanol production by scheffersomyces shehataeandsaccharomyces cerevisiae. *Biotechnol. Biofuels.* 7 63–79. 10.1186/1754-6834-7-63 24739736 PMC4005856

[B4] Gao X. Li K. Ma Z. Zou H. Wang J. (2020). Cucumber fusarium wilt resistance induced by intercropping with celery differs from that induced by the cucumber genotype and is related to sulfur-containing allelochemicals. *Sci. Hortic.* 271 109475–109485. 10.1016/j.scienta.2020.109475

[B5] Gu J. G. Lv X. Y. Hu D. D. Li S. G. Jiang R. B. (2008). Production of chlamydospores of *Trichoderma longbrachiatum* ACCC30150 and *Trichoderma harzianum* ACCC30371 by liquid fermentation. *Chin. J. Bio. Control* 24 253–256.

[B6] Jia H. H. Zhou H. Wei P. (2003). Advances in research and application of microwave mutagenesis. *Ind. Microbiol.* 33 46–50.

[B7] Jia K. Gao Y. H. Huang X. Q. Guo R. J. Li S. D. (2015). Rhizosphere inhibition of cucumber fusarium wilt by different surfactin- excreting strains of bacillus subtilis. *Plant Pathol.* 31 140–151. 10.5423/PPJ.OA.10.2014.0113 26060433 PMC4453995

[B8] José L. H. M. María I. S. P. Juan M. G. P. Jesús D. Q. V. Langarica H. R. G. (2015). Antibiosis of Trichoderma spp strains native to northeastern mexico against the pathogenic fungus macrophomina phaseolina. *Braz. J. Microbiol.* 46 1093–1101. 10.1590/S1517-838246420120177 26691467 PMC4704620

[B9] Ju X. Bowden M. Brown E. E. Zhang X. (2015). An improved x-ray diffraction method for cellulose crystallinity measurement. *Carbohydr. Polym.* 123 476–481. 10.1016/j.carbpol.2014.12.071 25843882

[B10] Karkachi N. E. Gharbi S. Kihal M. Henni J. E. (2012). Biological control of *Fusarium oxysporum* f.sp. lycopersici isolated from Algerian tomato by *Pseudomonas fluorescens*. *Bacillus cereus*, *Serratia marcescens* and *Trichoderma harzianum*. *Res. J. Agro.* 4 31–34. 10.3923/rjagr.2010.31.34

[B11] Khaledi N. Taheri P. (2016). Biocontrol mechanisms of trichoderma harzianum against soybean charcoal rot caused by macrophomina phaseolina. *J. Plant Prot. Res.* 56 21–31. 10.1515/jppr-2016-0004

[B12] Kim T. H. Lee Y. Y. (2005). Pretreatment and fractionation of corn stover by ammonia recycle percolation process. *Bioresour. Technol.* 96 2007–2013. 10.1016/j.biortech.2005.01.015 16112488

[B13] Kushwah P. Mishra T. Kothari V. (2013). Effect of microwave radiation on growth, enzyme activity (amylase and pectinase), and/or exopolysaccharide production in *Bacillus subtilis*, *Streptococcus* mutans, *Xanthomonas campestris* and *Pectobacterium carotovora*. *Br. Microb. Res. J.* 3 645–653. 10.9734/bmrj/2013/5036

[B14] Li J. F. Zhang S. Q. Shi S. L. Huo P. H. (2011). Mutational approach for N2-fixing and P-solbilizing mutant strains of *Klebsiella pneumoniae* RSN19 by microwave mutagenesis. *World J. Microb. Biot.* 27 1481–1489. 10.1007/s11274-010-0600-7 25187147

[B15] Li R. Shen Z. Z. Li S. Zhang R. F. Fu L. Deng X. H. (2016). Novel soil fumigation method for suppressing cucumber fusarium wilt disease associated with soil microflora alterations. *App. Soil. Ecol.* 101 28–36. 10.1016/j.apsoil.2016.01.004

[B16] Li Y. Q. (2001). Study on the screening high yield xylanase producing strain *Aspergillus Niger* by microwave mutagenesis. *J. Microwares.* 17 50–53.

[B17] Liu B. Zheng S. Ma X. Bo Y. Yue J. Dong W. (2015). Mutation breeding of extracellular polysaccharide-producing microalga *Crypthecodinium cohnii* by a novel mutagenesis with atmospheric and room temperature plasma. *Int. J. Mol. Sci.* 16 8201–8212. 10.3390/ijms16048201 25872142 PMC4425076

[B18] Liu Q. Li W. Ma Q. An S. Li M. Jameel H. (2016). Pretreatment of corn stover for sugar production using a two-stage dilute acid followed by wet-milling pretreatment process. *Bioresour. Technol.* 211 435–442. 10.1016/j.biortech.2016.03.131 27035475

[B19] Liu X. H. Xu C. H. Sun S. Q. Huang J. Zhang K. Li G. Y. (2012). Discrimination of different genuine danshen and their extracts by fourier transform infrared spectroscopy combined with two-dimensional correlation infrared spectroscopy. *Spectrochim. Acta Part A* 97 290–296. 10.1016/j.saa.2012.06.013 22771564

[B20] Mazinani S. A. Moradi F. Stuart J. A. Yan H. (2017). Microwave irradiation of PC3 cells at constant culture temperature alters the incorporation of BODIPY into cells and reduction of MTT. *ChemistrySelect* 2 7983–7986. 10.1002/slct.201701445

[B21] Osula O. Swatkoski S. Cotter R. J. (2015). Identification of protein sumoylation sites by mass spectrometry using combined microwave-assisted aspartic acid cleavage and tryptic digestion. *J. Mass Spectrom.* 47 644–654. 10.1002/jms.2959 22576878 PMC3470867

[B22] Qin Z. R. Wang Y. F. Guo J. Zhang D. D. Wang X. L. (2016). *A Selective Medium for Trichoderma Harzianum Culture. China: 201610586285.0.* Hefei: Hefei Dingtu Intellectual Property Agency Office.

[B23] Romuli S. Karaj S. Müller J. (2015). Influence of physical properties of *Jatropha curcas* L. seeds on shelling performance using a modified disc mill. *Ind. Crops Prod.* 77 1053–1062. 10.1016/j.indcrop.2015.10.014

[B24] Salgueiro A. M. Evtuguin D. V. Saraiva J. A. Almeida F. (2016). High pressure-promoted xylanase treatment to enhance papermaking properties of recycled pulp. *Appl. Microbiol. Biotechnol.* 100 1–9. 10.1007/s00253-016-7703-5 27383606

[B25] Sarkar S. Selvamurthy W. Gupta M. M. (2015). Biological consequences of microwave stress: implications for mutagenesis and carcinogenesis. *IETE Technical. Rev.* 14 153–163. 10.1080/02564602.1997.11416665

[B26] Segal L. Creely J. J. Martin A. E. Conrad C. M. (1959). An empirical method for estimating the degree of crystallinity of native cellulose using the x-ray diffractometer. *Text. Res. J.* 29 786–794. 10.1177/004051755902901003

[B27] Singh R. Krishna B. B. Kumar J. Bhaskar T. (2016). Opportunities for utilization of non-conventional energy sources for biomass pretreatment. *Bioresour. Technol.* 199 398–407. 10.1016/j.biortech.2015.08.117 26350883

[B28] Stuckey S. Storici F. (2013). Gene knockouts, in vivo site-directed mutagenesis and other modifications using the delitto perfetto system in *Saccharomyces cerevisiae*. *Method Enzymol.* 533 103–131. 10.1016/B978-0-12-420067-8.00008-8 24182920

[B29] Wang W. Wang X. M. Zhang Y. Yu Q. Tan X. S. Zhuang X. S. (2020). Effect of sodium hydroxide pretreatment on physicochemical changes and enzymatic hydrolysis of herbaceous and woody lignocelluloses. *Ind. Crops Prod.* 145 112145–112152. 10.1016/j.indcrop.2020.112145

[B30] Wu L. Q. Miao F. X. Gu H. K. Shang H. Z. (2012). Breeding of High-yield salinomycin-producing streptomyces albus strains by low energy N^+^ ion beam irradiation. *Agri. Biotechnol.* 1 55–56.

[B31] Xu T. T. Bai Z. Z. Wang L. J. He B. F. (2010). Breeding of D(-)-lactic acid high producing strain by low-energy ion implantation and preliminary analysis of related metabolism. *Appl.Biochem. Biotechnol.* 60 314–321. 10.1007/s12010-008-8274-4 18574566

[B32] Yan S. Wang S. Zhai Z. Chen X. Wu J. (2014). Mutation breeding of salt-tolerant and ethanol-producing strain *S.cerevisiae* H058 by low-energy ion implantation. *Adv. J. Food Sci. Technol.* 6 941–946. 10.19026/ajfst.6.136

[B33] Yang B. Dai Z. Ding S. Y. Charles E. W. (2011). Enzymatic hydrolysis of cellulosic Biomass. *Biofuels* 2 421–449. 10.4155/bfs.11.116

[B34] Yücel S. Karaçancı Ş Ay T. (2017). Activity and bioformulation of trichoderma harzianum for management of tomato diseases caused by soilborne pathogens. *Acta Hortic.* 1164 339–344. 10.17660/actahortic.2017.1164.43 34854763

