# Supplementary material for: Screening of Cucumber Fusarium Wilt Bio-Inhibitor: High Sporulation Trichoderma harzianum Mutant Cultured on Moso Bamboo Medium
Source: Front Microbiol. 2021 Dec 31;12:763006. doi: 10.3389/fmicb.2021.763006 (PMC8759106; doi:10.3389/fmicb.2021.763006)
Supplement: Supplementary file 1 [file Data_Sheet_1.docx]

Supplementary materials

Table 1 Inhibition rate and growth rate of 400-1-8, 400-2-16 and T334

| Samples | Inhibition rate | Growth rate (compared to the parental generation) | Growth rate (compared to the original strain) |
| --- | --- | --- | --- |
| Original strain | 63% | -- | -- |
| 400-1-8 | 69% | 9.52% | 9.52% |
| 400-2-16 | 74% | 7.25% | 17.46% |
| T334 | 78% | 5.41% | 23.81% |


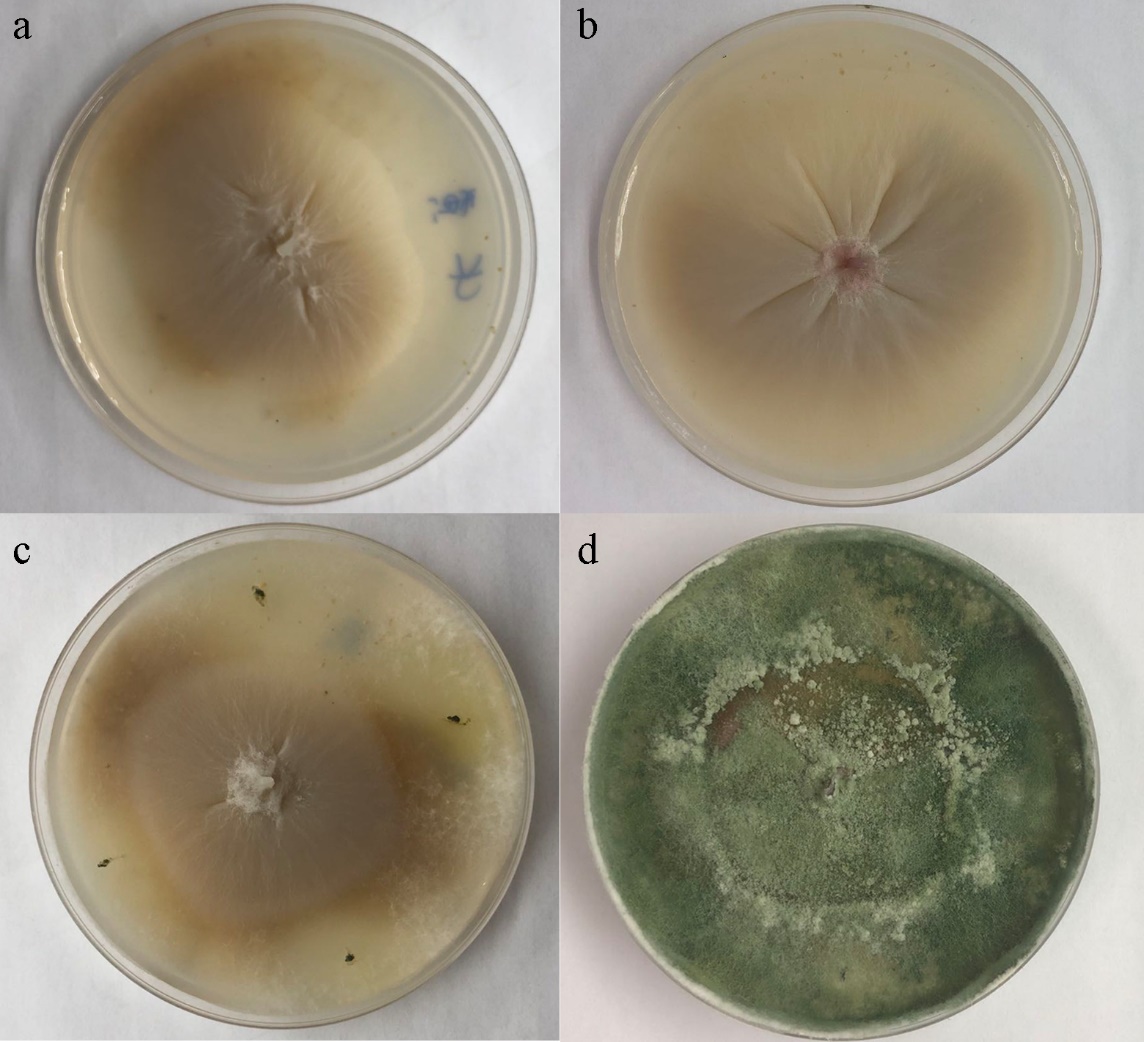


a: Single colony of *Fusarium oxysporum f. sp. cucumerinum* (CICC 2532) cultured for 48 h;

b: Single colony of *Trichoderma harzianum* T334 cultured for 48 h;

c: Antimicrobial experiment cultured for 48 h;

d: Antimicrobial experiment cultured for 144 h;

Table 2 Inhibition rate of *T. harzianum* on *Fusarium oxysporum f. sp. cucumerinum*

| Time | 48h | 72h | 96h |
| --- | --- | --- | --- |
| Inhibition rate (%) | 12% | 37% | 73% |
